# Microbiomes of the Sydney Rock Oyster are acquired through both vertical and horizontal transmission

**DOI:** 10.1186/s42523-022-00186-9

**Published:** 2022-05-19

**Authors:** Andrea Unzueta-Martínez, Elliot Scanes, Laura M. Parker, Pauline M. Ross, Wayne O’Connor, Jennifer L. Bowen

**Affiliations:** 1 Department of Marine and Environmental Science, Northeastern University, Nahant, MA 01908 USA; 2 School of Life and Environmental Sciences, The University of Sydney, Camperdown, NSW 2006 Australia; 3 Climate Change Cluster, University of Technology Sydney, Ultimo, NSW 2007 Australia; 4 School of Biological, Earth and Environmental Sciences, The University of New South Wales, Kensington, NSW 2052 Australia; 5 New South Wales Department of Primary Industries, Port Stephens Fisheries Institute, Taylors Beach, NSW 2316 Australia; 6 Present Address: Department of Organismic and Evolutionary Biology, Harvard University, Cambridge, MA 02138 USA

**Keywords:** Microbiome transmission, Animal microbiomes, Holobiont, Oyster larvae microbiomes, Sydney Rock Oyster, Vertical transmission, Horizontal transmission, Symbiosis

## Abstract

**Background:**

The term holobiont is widely accepted to describe animal hosts and their associated microorganisms. The genomes of all that the holobiont encompasses, are termed the hologenome and it has been proposed as a unit of selection in evolution. To demonstrate that natural selection acts on the hologenome, a significant portion of the associated microbial genomes should be transferred between generations. Using the Sydney Rock Oyster (*Saccostrea glomerata*) as a model, we tested if the microbes of this broadcast spawning species could be passed down to the next generation by conducting single parent crosses and tracking the microbiome from parent to offspring and throughout early larval stages using 16S rRNA gene amplicon sequencing. From each cross, we sampled adult tissues (mantle, gill, stomach, gonad, eggs or sperm), larvae (D-veliger, umbo, eyed pediveliger, and spat), and the surrounding environment (water and algae feed) for microbial community analysis.

**Results:**

We found that each larval stage has a distinct microbiome that is partially influenced by their parental microbiome, particularly the maternal egg microbiome. We also demonstrate the presence of core microbes that are consistent across all families, persist throughout early life stages (from eggs to spat), and are not detected in the microbiomes of the surrounding environment. In addition to the core microbiomes that span all life cycle stages, there is also evidence of environmentally acquired microbial communities, with earlier larval stages (D-veliger and umbo), more influenced by seawater microbiomes, and later larval stages (eyed pediveliger and spat) dominated by microbial members that are specific to oysters and not detected in the surrounding environment.

**Conclusion:**

Our study characterized the succession of oyster larvae microbiomes from gametes to spat and tracked selected members that persisted across multiple life stages. Overall our findings suggest that both horizontal and vertical transmission routes are possible for the complex microbial communities associated with a broadcast spawning marine invertebrate. We demonstrate that not all members of oyster-associated microbiomes are governed by the same ecological dynamics, which is critical for determining what constitutes a hologenome.

**Supplementary Information:**

The online version contains supplementary material available at 10.1186/s42523-022-00186-9.

## Background

Many host-associated microbiomes play critical roles in host fitness, physiology, immunity, behavior, and development [1]. Animals that are so closely linked to their associated microbes are no longer thought of as individual organisms, instead they are considered holobionts. This term encompasses the multicellular eukaryotic host and all of its associated microorganisms (bacteria, archaea, fungi, protists, and viruses), from obligate symbionts to transient microbes [2–5]. The genomes of all the members of the holobiont (host and microbiome genomes) can be thought of as the hologenome, which has been proposed as a unit of selection in evolution [2, 5, 6].

There are two main challenges raised against the hologenome concept. The first challenge suggests that if the microbial community and the host do not evolve together, then the hologenome is not real [7–9]. The term hologenome however, does not assume coevolution, it simply describes the genomes in the holobiont at a single point in time [10]. Second, questions exist regarding whether or not hologenomes are the primary unit of selection [9]. However, selection can occur at multiple levels of organization including selection on the host genome, the microbiome genomes, and the collective hologenome [2, 6, 10]. The key advance of the hologenome concept is that it promotes awareness that hosts and their associated microbes are tightly linked and that host-associated microbes can affect host fitness [1, 10, 11].

Understanding the evolutionary controls on hosts and their microbiomes across the host’s life cycle is essential to the proper interpretation of the hologenome. Larval development is a key evolutionary process and research is needed to understand how host-associated microbes shift through larval stages, how they shape animal development, and how they influence adult fitness. In vertebrates, the gut microbiome dynamically changes with early developmental milestones [12] and promotes the development of body organs and the immune system [13]. In marine invertebrates, single members of the microbiome can play a critical role in organ development [14]. However, unlike their vertebrate counterparts, it is unclear what role the host-associated microbial community plays in different developmental stages of invertebrates.

In addition to tracking the host microbiomes throughout larval development, it is also essential to disentangle intergenerational transmission modes. If some associated microbes and their genomes play important roles in the evolution of the host, they are likely transmitted between generations [6]. Transmission of associated microbes between generations is usually classified as either vertical, horizontal, or a combination of the two. In horizontal transmission, each new generation of the host acquires associated microbes from the surrounding environment, like the Hawaiian bobtail squid, *Euprymna scolopes,* and its critical symbiont, *Vibrio fischeri* [14]. In vertical transmission, associated microbes are passed down to the next generations on or in the gametes, as demonstrated in the marine sponge *Chondrilla australiensis* [15]. A variation on the definition of vertical transmission of microbes is referred to as maternal transmission, where microbes are transferred from the mother to the offspring, though not necessarily on or in the eggs. Examples of maternal transmission include the inoculation of the offspring through the birthing [16] and breastfeeding [17] processes in mammals.

To build an understanding of the role that microbes play in the health, and ultimately, evolution of organisms, we must determine the mechanism of intergenerational transfer and maintenance of microbial members of the holobiont. While there are examples of vertical transmission of obligate symbionts in some bivalves [18, 19], it is unknown how the complex communities of host microbes are transferred between generations and throughout different life cycle stages. Oysters are an excellent model system to study the role of horizontal and vertical transmission in marine invertebrates. They have been subject to numerous reproduction [20], development [21], and microbiome (reviewed in [22]) studies, which have revealed complex microbial communities in and on oyster larvae [23], and adult tissues [24–26]. Additionally, the well-established rearing methods of these commercial species allow for replicated and controlled characterization of host-associated microbes throughout multiple life stages. Oysters are also vital to communities and economies across the globe with their aquaculture production valued at 12 billion USD (United States Dollars) a year [27].

The oyster life cycle begins when sexually mature adults spawn their gametes into the water column, where fertilization and embryonic development take place. After fertilization, the first larval stage, the trochophore, is a free-swimming stage characterized by an apical sensory plate with a tuft of cilia [28]. The next larval stage, the D-veliger, is characterized by the development of the first shell and an organ called the velum that has a ring of cilia for swimming and eating [28]. In the next stage (umbo), larvae develop the protruding shape around the hinge, which gives oysters their distinctive form [28]. The last pelagic larval stage is the eyed pediveliger, characterized by the development of an eye spot and a “foot”, which is used to probe the substrate and find a suitable place to settle [28]. Once it has metamorphosed and settled, the oyster will no longer move and it is referred to as spat until it grows to adult size.

The Sydney Rock Oyster (*Saccostrea glomerata*) is native to eastern Australia where it forms a 40.9 million USD aquaculture industry [29, 30]. The microbiome of *S. glomerata* is known to respond to environmental change and possess an associated-bacterial community that is dependent on genotype [31]. Using *S. glomerata* as a model system, we tested whether oyster-associated microbes are passed down vertically, by conducting single parent crosses and tracking the microbiome from parent to offspring and throughout early larval stages using 16S rRNA gene amplicon sequencing. From each cross, we sampled adult tissues (mantle, gill, stomach, gonad, eggs or sperm), larvae (D-veliger, umbo, eyed pediveliger, and spat), and the surrounding environment (water and algae feed) for microbial community analysis. To determine whether there were horizontally acquired microbes associated with the different developmental stages of oysters, we assessed whether (1) there were microbial communities associated with each developmental stage that were unique to that stage across multiple family lines, and (2) the microbial communities at each larval stage were distinguishable from the surrounding water and food. To assess whether there was vertical transmission of microbes from parent to offspring we determined whether (3) there were microbes that persisted throughout all developmental stages (resulting in the formation of a core microbiome) and if (4) there was a proportion of the microbiome shared between parental gametes and their offspring.

## Methods

### Sydney Rock Oyster gamete and tissue collection

We purchased 20 adult Sydney Rock Oysters, *Saccostrea glomerata*, from an oyster farm in Port Stephens, New South Wales (NSW) (-32°45’S, 152°10’E) Australia (Holbert’s Oyster Supplies). These adults formed the parent generation used in our crosses and were approximately 1.5–2 years of age at the time of collection. We transported the oysters to the Department of Primary Industries (DPI) Port Stephens Fisheries Institute (PSFI), Taylors Beach, NSW, Australia, to strip spawn the oysters and make single parent crosses. All seawater used in subsequent experiments and was collected from the estuary adjacent to PSFI (− 32° 44′ 39.2568″ S, 152° 3′ 15.8394″ E) and had a salinity of 34 PSU (practical salinity unit). Seawater was filtered through two 1 μm filter bags and stored in 34,000 L polyurethane tanks onsite.

Once we identified an oyster as a competent (ready to release gametes) female, we removed it from its shell and put it in a sterile petri dish. We scored the gonad tissue with a sterile razor multiple times on both sides and rinsed it with filtered seawater (1 μm filter) to collect the eggs in a sterile glass beaker. Roughly 2000 eggs were pipetted out of the beaker and passed through a stack of sterile, single use cell strainers (pluriStrainer®, pluriSelect Life Science, Leipzig, Germany) to separate debris and capture the eggs. The collection cell strainer (20 μm) with the eggs was rinsed thoroughly with sterile seawater (seawater that was filtered through two 1 μm filter bags and then autoclaved) to remove loosely associated microbes. The cell strainer with the eggs was put in a sterile Whirl–pak (NASCO WHIRL–PAK®, USA) bag, flash-frozen with liquid nitrogen, and stored at − 80 °C until DNA extraction. The remaining eggs in the initial collection beaker were then used for fertilization. We repeated this procedure for each female (*n* = 5).

When we identified an oyster as a competent male, we removed it from its shell and placed it in a sterile petri dish. We scored the gonad tissue with a sterile razor multiple times on both sides, rinsed it with sterile seawater, and, to remove impurities, filtered the sperm through a single-use, sterile 10 μm pluriStrainer cell strainer and collected the sperm in a sterile falcon tube. Twenty-five μL of sperm were pipetted into a cryovial, flash-frozen in liquid nitrogen, and stored at − 80 °C until DNA extraction. We used the remaining sperm from each male (*n* = 5) to fertilize the eggs of a specific female, to derive single parent crosses.

Lastly, we collected tissues from the adults used for the crosses. After each adult was rinsed to collect the eggs or sperm, the oysters were transferred back into their shells until the crosses were completed. We then collected mantle, gill, stomach, and gonad tissues for each adult oyster. All of the tissues were thoroughly rinsed with sterile seawater, flash-frozen in liquid nitrogen, and stored at − 80 °C until DNA extraction.

### Crosses and larval rearing

We set up five independent single-parent crosses. Fertilization took place in 20 L buckets following the methods of Parker et al. [32]. Buckets were sterilized using Virkon S (Antec International) and filled with 1 μm filtered seawater set at 23 °C. Approximately one million eggs from each female were placed into buckets, sperm was then added incrementally, the mixture was homogenized gently and then a subsample was checked under a light microscope (*Leica* 200x). This process was repeated until a ratio of 5 sperm per egg was observable in a subsample of the mixture. The gametes were then allowed to rest for 30 min. Fertilized gametes from each cross were then transferred into three 200 L polyethylene larval rearing tanks to create three independent replicates for each single parent cross (*n* = 15 tanks). Tanks were sterilized using Virkon S and filled with filtered (1 μm) seawater at 23 °C. Larval feeding began after roughly 16 h, with the appearance of the first D-veliger larvae. We fed the larvae twice a day with an algal diet of 50% *Chaetoceros muelleri*, 25% *Diacronema lutheri* and 25% *Tisochrysis lutea.* Algal concentrations started at 1 × 10^4^ cells per mL at the beginning of the experiment and ended at 1.16 × 10^5^ cells per mL at the completion of the experiment as the larvae increased in size. We performed water changes every second day throughout the experiment and followed rearing protocols optimal for *S. glomerata* larvae that are described in detail in O’Connor et al. [33]. After 33 days the larvae reached the pediveliger stage and showed signs of readiness to settle (eye spot, 300 μm shell length, protruding foot, crawling). We then dosed them with epinephrine to induce settlement, as is commonly done in the hatchery production of this species [33].

### Larvae collection

For each single parent cross replicate, we collected multiple stages of larval development: day 1 and day 3 (D-veliger), day 15 (umbo), day 29 (eyed pediveliger), and day 34 (spat). We collected roughly 2,000 individuals from each tank at each larval and spat stage and passed them through a stack of sterile, single-use pluriStrainer cell strainers to separate debris and capture the larvae. The collection cell strainers (40 μm, 70 μm, and 100 μm) with the larvae were rinsed thoroughly with sterile (autoclaved) seawater to remove loosely associated microbes, placed in sterile Whirl–pak bags, flash-frozen with liquid nitrogen, and stored at − 80 °C until DNA extraction. Additionally, we collected seawater and algae feed samples from each tank for microbial community analysis at each of the larval stages. We filtered 500 mL of tank water through a 0.22 μm Sterivex filter. Separately, we pipetted 1 mL of the larvae feed mix into a cryovial. Both water and feed mix were collected in triplicate, flash-frozen, and stored at − 80 °C until DNA extraction.

### Nucleic acid preparation and sequencing

We extracted nucleic acids from the adult tissues, gametes, larvae, and environmental samples using the DNeasy PowerLyzer PowerSoil kit (Qiagen, Valencia, CA USA) following the manufacturer’s protocol. To amplify the V4 region of the 16S rRNA gene in triplicate 25 μL polymerase chain reactions (PCR), for all samples we used 5 PRIME Hot Master Mix (Quanta Bio, Beverly, MA USA) and the primers 515FY: 5′TATGGTAATTGTGTGYCAGCMGCCGCGGTAA 3′ [34] and 806RB: 3′ AGTCAGTCAGCCGGACTACNVGGGTWTCTAAT 5′ [35]. The thermocycler conditions were as follows: a 3 min hot start at 94 °C followed by 35 cycles of 94 °C for 45 s, 50 °C for 60 s, and 72 °C for 90 s. The final extension step was 72 °C for 10 min. After checking the triplicate PCR product and negative controls on a gel to ensure the product matched the target size of ~ 390 bp and that there was no contamination, we purified and size selected the PCR products using Agencourt AMPure Magnetic Beads (Beckman Coulter, Brea, CA USA), and resuspended them in 20 μL of nuclease-free water. We then ligated Illumina paired-end adapters with unique Nextera XT v2 indexes to 2 μL of 16S rRNA amplicons using 8 cycles of PCR. The thermocycler conditions were as follows: a 3-min hot start at 95 °C followed by 8 cycles of 95 °C for 30 s, 55 °C for 30 s, 72 °C for 30 s, and a final extension step at 72 °C for 5 min. We then purified and size selected the PCR products using Agencourt AMPure Magnetic Beads, and resuspended them in 20 μL of nuclease-free water. We quantified our libraries using the Quant-iT PicoGreen dsDNA Assay Kit (Invitrogen, Carlsbad, CA USA) and pooled them at equimolar concentrations. After confirming library size on an Agilent 4200 TapeStation (Agilent Technologies, Santa Clara, CA USA), we quantified the library using a KAPA library quantification kit (Roche Sequencing Solutions Inc., Pleasanton, CA USA) and sequenced our library on an Illumina MiSeq with 2 × 250 V2 sequencing chemistry at the Tufts University Core Sequencing Facility.

### Procedural controls

We collected procedural negative controls that were carried through library preparation and sequencing. While sampling, we collected negative controls for (1) all cell strainer sizes used to sample gametes and larvae and (2) for the Sterivex filters used to sample seawater microbes. During the library preparation process, we included DNA extraction controls with every batch, and PCR amplification controls. Additionally, we sequenced three replicates of a mock community (ZymoBIOMICS™ Microbial Community DNA Standard, Zymo Research, USA), with known theoretical relative abundances of 10 species, as a positive control. Additional file 1: Fig. S1 illustrates that our mock community replicates were highly consistent with their expected composition.

### Sequence analysis

We used the DADA2 (v1.7.0) workflow with default parameters [36], implemented in R Studio (v4.0.0), to quality-filter, merge paired-end reads, remove chimeric sequences, group the sequences into amplicon sequence variants (ASVs), and assign taxonomy against the Silva database (version 132; [37]). Initial processing of the ASV table was conducted using the Phyloseq package [38]. We identified potential procedural and reagent contaminants using the decontam package based on either the frequency of each ASV as a function of the input DNA concentration or the prevalence of each ASV in true samples compared to the prevalence in negative controls [39]. The Decontam package successfully removes 74–91% of contaminants when the source of contamination is not well defined [40]. We assessed the composition of the mock communities to ensure they agreed with the theoretical composition (Additional file 1: Fig. S1). We also filtered out singleton ASVs and ASVs identified as mitochondria, chloroplasts, Eukaryota, and Archaea, which accounted for less than 3% of our data set. Samples with less than 1000 reads after quality filtering were removed from the data set (*n* = 13 of 192). Rarefaction analyses confirmed that the sequencing coverage was sufficient to get the vast majority of the bacterial diversity in all oyster tissue samples (Additional file 1: Fig. S2). To account for uneven sequencing depths across samples, we (1) transformed our data to proportions by dividing the reads for each ASV in a sample by the total number of reads in that sample, as previously recommended [41–45] to conduct β diversity analyses, and (2) normalized reads by converting ASV abundances to Z-scores before running Random Forrest classification models. The rest of our statistical analyses relied on presence/absence data of samples that were sequenced deeply enough to have representative diversity.

### Statistical analysis

To test our first two hypotheses of whether (1) different larval stages harbor distinct microbial communities and (2) larvae microbiomes differ from water and algae microbiomes, we focused on β diversity and computed a Sorensen-Dice dissimilarity matrix using the vegdist function in Vegan [46]. We used this dissimilarity matrix to run three independent permutational multivariate analysis of variance (PERMANOVA), with larval stage, environment, and family as factors all with 999 permutations using the adonis2 function in Vegan [46]. We ran post-hoc pairwise comparisons, with Bonferroni corrected *P-*values, to compare larval stages to each other, to the gametes, and to their surrounding environment, using the custom function pairwise.adonis (https://github.com/pmartinezarbizu/pairwiseAdonis; [47]). We also tested for homogeneity of multivariate dispersions using betadisper, and ran post-hoc pairwise comparisons using the permutest.betadisper function in Vegan [46]. To visualize β diversity of the different larval stages, gametes, and their surrounding environment, we used non-metric multidimensional scaling (NMDS) plots of the Sorensen-Dice dissimilarities using the metaNMDS package in Vegan [46]. We carried out our statistical analyses following the recommendations of the Guide to Statistical Analysis in Microbial Ecology [48].

To further investigate the microbial communities associated with different larval stages, we performed a network analysis using Gephi [49] to determine the distribution of ASVs (relative abundance > 0.001). Gephi uses a force-directed graph algorithm to visualize the network (ForceAtlas2). We then conducted modularity analysis on the network (Louvain), and ran a Random Forest Classification model using 10,001 trees on a feature table containing ASVs that were in more than 20% of the samples (179 ASVs total) to find the ASVs that best describe each larval stage (randomForest R package; [50]). Model performance was confirmed by examining the out-of-bag error rate and we performed leave-one-out cross-validation with 999 permutations in the caret R package [51].

To test our second two hypotheses regarding (3) whether there was a portion of the microbiome (a core) that persisted across all life cycle stages and (4) whether larvae shared a portion of their microbiome with their parent gametes, we focused on oyster-specific ASVs. We used set theory functions in R and a more conservative relative abundance cut off of 0.01 to find ASVs present in oyster samples that were not detected in the oyster environment (tank water and algae). These oyster-specific ASVs were then used to investigate whether any microbes persisted across all life cycle stages. To do this we performed a core analysis with a relative abundance > 0.01 and prevalence > 0.5, as defined in previous studies [52], using the core_members function in the microbiome R package [53]. We also used the oyster-specific ASVs to investigate whether there was a portion of the larvae microbiome shared with parent gametes. We used set theory functions in R and performed pairwise comparisons of all parent and offspring pairs to find the percentage of ASVs in the offspring that were shared with their parent eggs and sperm. To accompany this, we performed a Mann–Whitney U test to compare whether offspring shared more ASVs with their parent eggs or sperm. All figures were created using ggplot2 [54].

## Results

**Fig. 1:**
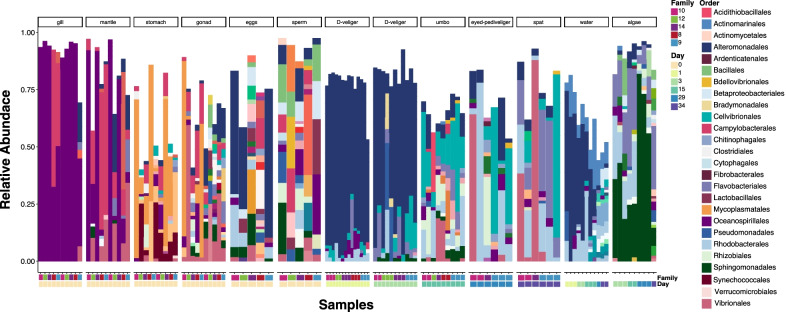
Stacked bar plot of the relative abundance of bacterial orders comprising microbial communities associated with oyster tissues (gill, mantle, stomach, gonad, gametes and larvae) and their environment (water and algae). Relative abundance was calculated within each sample and ASVs that made up less than 1% of the sample were excluded

**Fig. 2:**
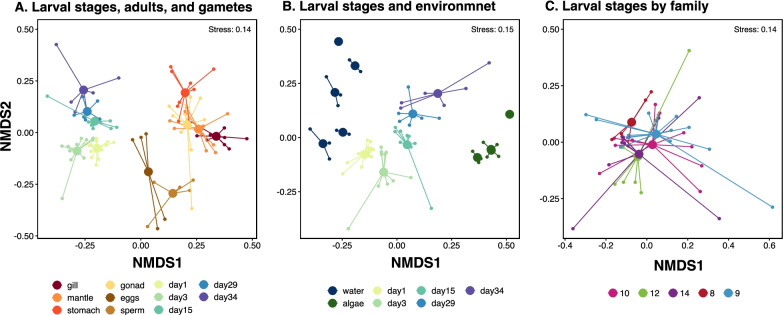
Non-metric multidimensional scaling (NMDS) plots of Sorensen-Dice dissimilarities of microbial communities associated with oyster larvae, gametes, and their environment. Colored by **A** larval stage day, adult tissues and gametes, **B** larvae stage day and environment, and **C** family. Group centroids are defined by the mean dissimilarities for each group

We found that each larval stage harbored compositionally distinct microbiomes that were different from their surrounding environment (Fig. 1). Non-metric multidimensional scaling (NMDS) ordination plots of the Sorensen-Dice dissimilarity indices showed distinct larvae-associated microbial communities that clustered by adult tissue types, larval stage, and gametes (Fig. 2a), and that were also distinct from the tank water and algae (Fig. 2b). Furthermore, permutational multivariate analysis of variance (PERMANOVA) models showed a significant (*P* < 0.001) effect of larval stage on microbial community composition (Additional file 1: Table S1), with pairwise significant differences (*P* < 0.05) between all pairs of larval stages except between day 29 (eyed pediveliger) larvae and day 34 spat (*P* > 0.05; Additional file 1: Table S1). Generally, adult tissues were different from larval stages, gametes, and each other (*P* < 0.05). PERMANOVA models also showed that larval microbiomes were distinct from tank water and algae feed (*P* < 0.001) at all larval stages (Additional file 1: Table S2). When considering all larval stages, individual families did not have distinct microbiomes (Fig. 2c).

We found high inter-individual variability throughout all life stages, however, this variability was especially noticeable in sperm, eggs, and older larvae (Fig. 1). We observed changes over time in the larvae and water microbiomes, with the bacterial order Alteromonadales having greater relative abundance in younger larvae and decreasing in abundance over time (Fig. 1). Many of the Alteromonadales ASVs that were highly abundant at earlier larval stages were also abundant in water samples (Additional file 1: Fig. S3), although their relative abundance patterns in early larval stages do not resemble the relative abundance patterns in the water samples. There were also Alteromonadales ASVs that were unique to oyster tissues and not detected in the water or algae samples (Additional file 1: Fig. S3), possibly these ASVs were present in such low abundances in water or algae samples that we did not detect them using our sampling methods. Other bacterial orders like Cellvibrionales increased in abundance through the umbo stage and remained abundant in some families but not others, through the spat stage (Fig. 1).

**Fig. 3:**
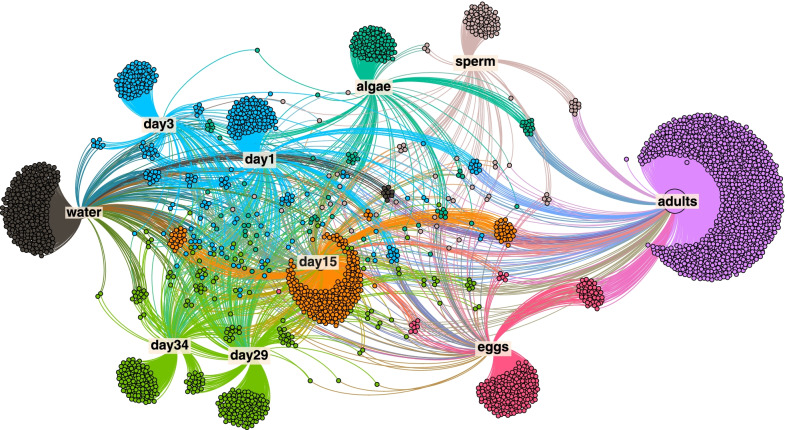
Force directed network of the ASVs in our data set with a relative abundance > 0.001 (*n* = 4420). Every dot identifies an ASV and the edges connect the ASVs to the sample type they were found in. Colors represent different modules identified in modularity analysis of the network

We observed compositional differences in the microbial community depending on larval stages, gamete type, and the environment. However, when examining specific ASVs, we found many ASVs that were shared among multiple larval stages. Network analysis allowed us to visualize the distribution of ASVs among larvae, adults, seawater, and algae samples. ASVs unique to a single group accounted for the largest fraction of ASVs (3724 of 4420), while ASVs shared by all groups were the smallest fraction of the total (127 of 4420). Modularity analysis indicates regions of the network that are more closely connected and in this network we identified 8 such modules (Fig. 3). The modularity analysis revealed that earlier larval stages, D-veligers (day 1) and D-veligers (day 3) form one module indicating that they have many ASVs in common, similarly, later larval stages day 29 (eyed pediveligers) and day 34 (spat) were also grouped in one module. All other sample types (adults, eggs, sperm, umbo, water, and algae) were identified as unique modules indicating their distinctness (Fig. 3).

**Fig. 4:**
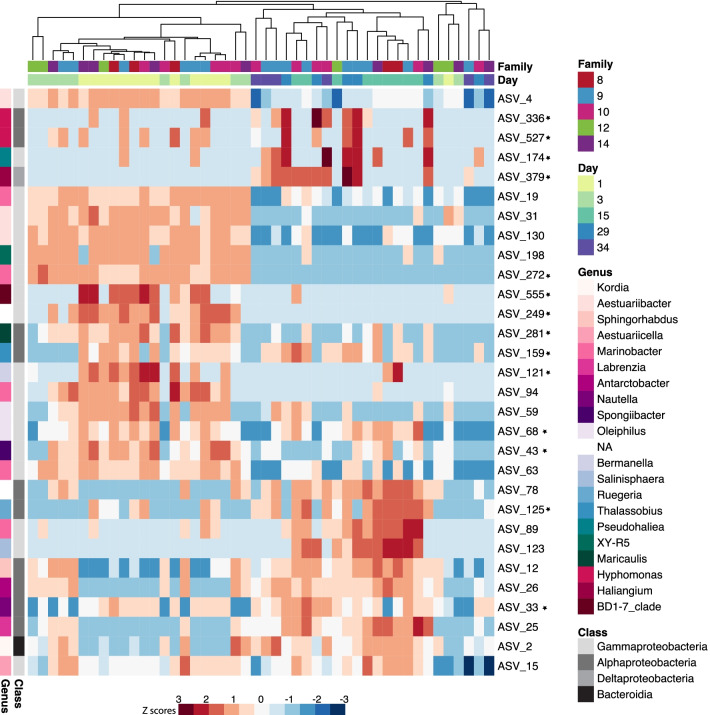
Heat map of the 30 ASVs that are the most important contributors to a Random Forest classification model that was trained to predict larval stage from microbial community composition. The heat map shows the relative abundance of the ASVs in samples of the different larval stages (Day), samples are clustered using Sorensen-Dice dissimilarity distances. More detailed taxonomic information can be found in Additional file 1: Table S4. Stars denote ASVs that were not detected in tank water and algae samples with a relative abundance greater than 1%

The random forest model that determined which ASVs best describe each larval stage, correctly classified the larvae microbial communities as belonging to the different larval stages 75.91% of the time with a 26.09% out-of-bag error rate. Older larval stages were more difficult for the model to predict than younger larval stages, 100% of day 1 (D-veligers), 90% of day 3 (D-veligers), and 88% of day 15 (umbo) larvae were classified correctly, while 0% day 29 (eyed pediveligers), and 33% of day 34 (spat) were classified correctly. Leave-one-out cross-validation confirmed model performance, with a Cohen’s kappa statistic of 71.72%. Of the top 30 ASVs that best describe each larval stage, 18 of them were Gammaproteobacteria, 10 were Alphaproteobacteria, one was a Deltaproteobacteria, and one was a Bacteroidia (Fig. 4).

**Fig. 5:**
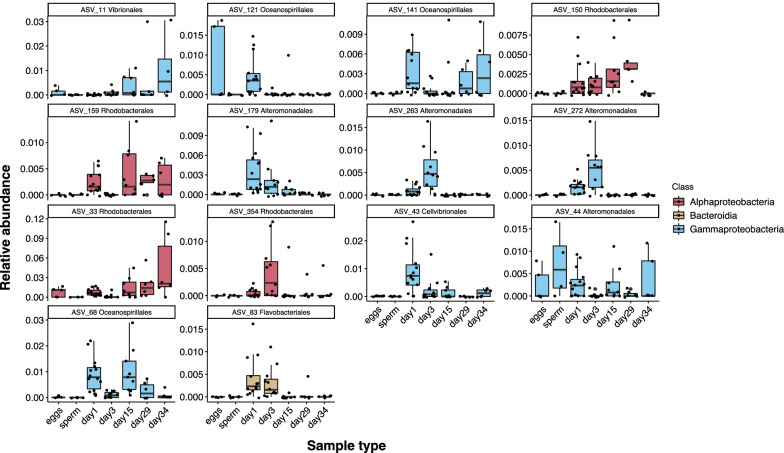
Box and whisker plots of the relative abundances of ASVs identified as core members (present at greater than 1% abundance in more than 50% of samples) in the eggs, sperm, and all larval stages. This analysis excluded all ASVs detected in tank water and algae samples with a relative abundance greater than 1%. More detailed taxonomic information can be found in Additional file 1: Table S5

**Fig. 6:**
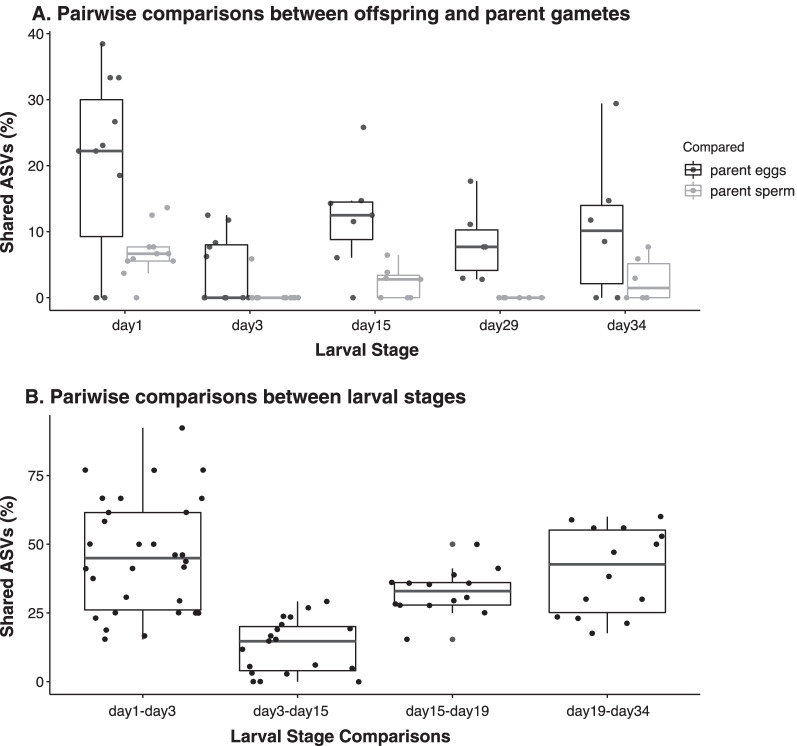
Box and whiskers plots of the percentage of ASVs shared **A** between each larval stage and their parent eggs and sperm, and **B** between adjacent larval stages. This analysis excluded all ASVs detected in tank water and algae samples with a relative abundance greater than 1%

We found 385 ASVs that were unique to oyster adult and larvae tissues and were not detected in the environment (tank water and algae). A core analysis of these oyster-specific microbes, indicated ASVs that were maintained across many, if not all, life cycle stages within each family. We found a total of 14 core ASVs including, ASV_33 and ASV_11 belonging to the Orders Rhodobacterales and Vibrionales respectively, in the eggs and all larval stages with increasing relative abundance through time (Fig. 5). ASV_44 in the Order Alteromonadales, was observed in the eggs and sperm of nearly all families and persisted in the larval stages of nearly all families. Additionally, we found that offspring shared significantly more ASVs with their parent eggs than with their parent sperm (Mann–Whitney, day 1: *P* = 0.05, day 3: *P* = 0.04, day 15: *P* = 0.02, day 29: *P* = 0.002, day 34: *P* > 0.05; Fig. 6A; Additional file 1: Table S6), and that a portion of the microbiome was conserved between larval stages as they grew older (Fig. 6B).

## Discussion

Elucidating how microbes are transferred across generations and maintained across the larval cycle is critical to our understanding of the role of microbes in the evolution of animals. We tracked the presence of microbes across parent gametes, larval stages, and their surrounding environment (i.e., tank water and algae feed) and identified microbes that are potentially transferred vertically and, based on existing literature, may infer benefits to the host. We found that early oyster larvae shared a portion of their microbiome with parent eggs and sperm suggesting the possible transmission of microbes from their parents, primarily via eggs. Subsequent development of the larval microbiome was then consistently parallel across families, suggesting horizontal transmission of larval stage-specific microbes from their environment. These findings support the holobiont concept by demonstrating that microbes were consistently associated with gametes and specific larval stages across five independent families. We showed that (1) different developmental stages had distinct microbiomes, and (2) the microbial communities at each larval stage were distinguishable from the surrounding water and food. We also assessed whether there was vertical transmission of microbes from parent to offspring by determining that (3) there were microbes that persist throughout all developmental stages and that (4) there was a proportion of the microbiome shared between offspring and their parental gametes, particularly the eggs. Thus, our findings provide evidence for both horizontal and vertical transmission strategies being used to transfer the complex communities of microbes across larval stages of a broadcast spawning marine species. We also highlight key taxa that are conserved across parent, offspring, and developmental stages suggesting that they potentially play important roles in oyster fitness.

### Larvae microbiomes vary with host developmental stages

Animal development is influenced by and, in some cases, depends on their associated microbiome. Like many marine invertebrates, oyster life history involves distinct planktonic and sessile phases. Our results support the hypothesis that oyster larvae-associated microbial communities change across developmental stages, with taxonomic membership shifting in consistent ways across multiple family lines throughout life history stages from eggs to spat. These consistent shifts suggest specific life-cycle stage selection of microbes from the environment via horizontal transmission. This is consistent with patterns observed in bacterial communities of fish larvae [55, 56], shrimp larvae [57] and Eastern oyster larvae [58]. The specificity of microbial communities at each larval stage suggests that the host environment shapes a portion of the microbial community in planktonic marine larvae through horizontal acquisition of microbes.

When we clustered our larvae microbiome samples based on shared taxa (Fig. 4); earlier larval stages generally clustered together and were separate from the late larval stages. This partitioning between earlier and later larval stages indicates a gradual progression in the microbial community composition between adjacent stages. This pattern has also been observed in sea urchin larvae, where their bacterial communities clustered based on shared taxa by sequential developmental stages [59]. It was surprising that we did not observe more abrupt shifts between major developmental milestones such as when larvae begin feeding or metamorphose into spat. This may be because during metamorphosis oyster larvae reabsorb larval organs, such as the foot and velum. The spat may have retained, instead of shed [60], a portion of the eyed pediveliger microbial community. There may also be a lag between metamorphosis and the time it takes for a new spat-specific microbial community to assemble.

Some of the ASVs identified as important contributors to the microbiomes of day 1 and day 3 larvae (D-veliger; ASV_4, ASV_31, ASV_130) belong to the class Gammaproteobacteria, family *Alteromonadaceae* (Fig. 4). ASV_44, a member of the core microbiome (Fig. 5) was also a member of the *Alteromondaceae* and was prevalent in the eggs, and particularly in the sperm, as well as in several subsequent larval stages, suggesting it may be a key component of vertical transmission. Members of this family have also been identified as dominant in D-veliger larvae of Pacific oysters [61]. Potential beneficial roles of *Alteromonadaceae* include protection against pathogens [62] and provision of fixed nitrogen to their animal host [63]. The protection and nutritional properties of this bacterial family could confer benefits to the host during the early more vulnerable life stages of oyster larvae. Additionally, members of this family were detected in oyster tissues but not in their environment (Additional file 1: Fig. S3). Possibly these ASVs were present in tank water and algae in such low abundances that we were unable to detect them using our sampling methods. Nonetheless, considering their notable abundance in the D-Veliger stage and their prevalence in other oyster microbiomes suggests more effort should be directed at understanding their role in oyster larval development. Some ASVs that were important at later larval stages, day 15 (umbo), day 29 (eyed pediveliger) and day 34 (spat; ASV_26, ASV_33, ASV_125), were members of the class Alphaproteobacteria, family *Rhodobacteraceae* (Fig. 4)*.* Bacteria of this family are widespread in marine ecosystems and are commonly associated with marine eukaryotes [64]. Many host-associated *Rhodobacteraceae* strains produce vitamin B_12_ [64, 65], which can radically alter gene expression and can accelerate development and reduce mortality in invertebrate hosts [66, 67]. Members of this family were also identified in high abundance in pre-settlement Pacific oyster larvae [61], and they may play a key role in the B_12_ supply of late larval stages. Additionally, host-associated *Rhodobacteraceae* strains produce an assortment of extracellular signaling compounds [65] that may be involved in controlling the physiological activities of host-associated bacterial communities. The nutritional and signaling properties of this family may be important to pre-settlement larval stages as they prepare to metamorphose.

### Larvae microbiomes are different from their environment

In addition to demonstrating a distinct and consistent progression during larval stages, our findings also indicate that larvae microbiomes were not merely a reflection of their surrounding water and food. These data are consistent with previous studies of marine invertebrate larvae in wild [68] and hatchery [23, 57, 58, 69] settings. Despite maintaining a distinct microbiome, the environment plays an important role in the initial establishment of host-associated microbes [13, 16]. We found that seawater microbes seem to be more important in influencing the microbiomes of earlier larval stages (D-veligers) than later larval stages (eyed pediveligers and spat) (Fig. 1; Additional file 1: Fig. S3). Similarly, shrimp larval microbes were influenced at earlier but not later larval stages by the rearing water microbiomes [57]. Our findings suggest that microbes in the environment may be important to the establishment of the microbiomes of earlier oyster larvae stages. This is of relevance to the oyster aquaculture industry and oyster conservation efforts because the D-veliger stage may be the most vulnerable to environmental microorganisms.

The influence of diet on marine larvae microbiomes is inconsistent in the literature. Some studies found a strong influence of diet-associated microbes on larval stages [70], while others found no effect of food microbes on larvae microbiomes [55]. Our findings agree with the latter, as we found that algae feed did not contribute significantly to oyster larvae microbiomes. It is possible that algae feed did not contribute to shifts in the larvae because we maintained the same algal diet throughout the experiment. By contrast, studies that change the feed source depending on the larval stage had larvae microbiome changes that coincided with changes in diet [70]. Further work is needed to determine the contribution of diet to the formation of aquatic larvae microbiomes. Experiments that focus on a single larval stage and have different food types as a factor would be particularly illustrative. Divergence from algal and tank water microbial communities suggests that oyster larvae select at least part of their associated microbial communities, rather than mirror the communities present in their surroundings, as seen in other invertebrates [71]. The consistent patterns of microbes selected at each larval stage across all oyster family lines suggest they are acquired via horizontal transmission.

### Some microbes persist throughout all developmental stages

We found that there was a core microbiome, not detected in the environment, that was persistent across multiple life cycle stages, including gametes, which suggests this may be vertically transmitted. The ASVs that were consistently found throughout the larval cycle and in different families include microbes with known symbiotic features and functional capabilities. Two of the ASVs identified in the core microbiome started in low relative abundances in the eggs and slowly increased in relative abundance through larval stages, resulting in a higher abundance in the spat stage relative to the eggs (Fig. 5). One of the ASVs that followed this pattern was ASV_33, belonging to the order Rhodobacterales, family *Rhodobacteraceae*, genus *Nautella*. This genus of bacteria is thought to be deposited into the egg cases of cephalopod mollusks where they are hypothesized to play a role in egg defense [72, 73]. Host-associated members of this family also produce vitamin B_12_ [64, 65] which can play a role in invertebrate development [66, 67]. This ASV was identified by both our core analysis (Fig. 5) and the Random Forest model that determined which ASVs best describe each larval stage (Fig. 4), the maintenance and abundance patterns of this ASV suggest that its functional importance should be investigated due to its prevalence on Sydney Rock Oyster larvae.

Another core ASV with a unique relative abundance pattern was ASV_44. This ASV had the highest mean relative abundance in sperm samples relative to its abundance in eggs and larval stages, and it belongs to the order Alteromonadales, genus *Pseudoalteromonas*. Strains from this genus were previously isolated from oyster hemolymph and shown to have antibacterial activity against gram-negative bacteria including *Vibrio* strains that are pathogenic to oysters [74]. It is possible that *Pseudoalteromonas* could protect sperm from detrimental bacteria that cause sperm agglutination and reduce sperm success [75]. This biologically active genus may also help create an extracellular environment in the seminal plasma suitable for sperm activation success [76, 77]. Although previous studies have found relationships between microbial community structure and sperm fertilization success [78, 79] further work is necessary to determine the function of sperm-associated bacteria.

Some ASVs identified in the core analysis had patchy relative abundance patterns across life history stages, such that they were abundant at some larval stages but not at others, possibly indicating their importance at a specific larval stage. For example ASV_141, ASV_121, and ASV_68, which had patchy abundance patterns, belong to the order Oceanospirillales (Fig. 5). Members of this order are commonly found in association with oyster larvae [23] and adults [22], and are symbiotic with the gills of many bivalves [80–82]. Additionally, they are known for their capacity to break down organic compounds in the environment and their abundance in crude-oil containing seawater [83, 84]. Their symbiotic capabilities with bivalves indicate that Oceanospirillales may confer beneficial effects to their larvae host and may be more useful at some larval stages than others. Within this order, ASV_121, family *Saccharospirillaceae*, genus *Bermanella,* was particularly interesting because of its high abundance in the eggs and first larval stage. Members of this genus produce poly-β-hydroxybutyrate (PHB) [85]. PHB is an energy storage compound produced by multiple types of bacteria [85–87] and has been demonstrated to increase survival and growth in various marine organisms [88–90] and protect marine larvae from infection by *Vibrio* pathogens [91, 92]. It is possible that *Bermanella* could have a beneficial association with oyster eggs and larvae by inhibiting pathogens and promoting growth.

### Larvae share a portion of their microbiomes with parent gametes and adjacent larval stages

We found that oyster larvae shared a higher percentage of ASVs with their parent eggs than sperm, suggesting that maternal contributions to larvae were more important than paternal contributions. This finding is not surprising considering that maternal provisions of microbes to offspring via the eggs is widespread in marine and terrestrial animals, with examples spanning multiple phyla ranging from Porifera, Molluska, Arthropoda, and Chordata (reviewed in [93]). Larvae shared a relatively small percentage of their microbes with their parent sperm, which suggest a small paternal contribution. This finding is also consistent with other examples of sperm-mediated vertical transmission in the marine environment [15]. Although there are multiple examples of biparental transmission of microbes in invertebrates [15, 94, 95] our study documents biparental modes of vertical transmission in oysters.

We also found that adjacent larval stages shared a relatively high percentage of their microbiomes (Fig. 6B). This suggests that, despite family line, larvae acquired the same microbes from the environment and conserved a portion of them throughout the gradual succession of microbiomes during development. Gradual succession patterns in other marine invertebrate larvae have been previously documented [57, 59]. These findings suggest that from early to late stages of larval development some members of their microbiome persist, some are lost, and some are gained along the way. The microorganisms that persist alongside the ones that are gained and carried forward are likely important to host fitness.

## Conclusions

The hologenome theory of evolution relies on the assumption that parents consistently transfer beneficial microbes to their offspring. We tested this assumption by using Sydney Rock Oysters as a model and conducting single parent crosses to track the microbiomes from parents to offspring. We characterized the succession of oyster larvae microbiomes from gametes to spat. We found that oyster-associated bacterial communities varied with host developmental stage, differed from their environment microbiomes, and had select members that persisted across multiple life stages. We also demonstrate that both vertical and horizontal transmission routes are possible for the complex communities of microbes associated with broadcast spawning marine invertebrates and that not all members of the oyster microbiome are governed by the same ecological dynamics. The functional importance, evolutionary significance, and mechanisms that drive changes throughout life history stages of oyster-associated microbial communities require additional investigation. Future experiments involving combined molecular microbiological (e.g. metagenomics) and geochemical approaches that can link microbial identity with metabolic activity are needed to explicitly test the functional relevance of host-associated microbiomes that are both vertically and horizontally transmitted across generations.

While our experiment does not directly test the hologenome theory of animal microbiomes, our results contribute to the ongoing conversation of this heavily debated topic (reviewed in [2, 10]). Some of our results are relevant to the assumptions posed by the hologenome theory of evolution. To determine if the hologenome is a level of selection, one of the critical assumptions is of consistent microbiome transmission from one holobiont generation to the next [2]. Theoretically, ‘consistent microbiome transmission’ is defined as offspring microbiota being more similar to the microbiomes of their parents than to those of other unrelated adults in the population [2]. Our findings, however, suggest that vertically and horizontally transmitted microbes were not different depending on oyster family, but they were rather consistently replicated across oyster families. This means that different oyster families were transferring the same microbes to their offspring. Our finding is consistent with a previous study that showed that vertically transmitted microbes were not faithful to a single marine sponge species [96]. While our results suggest that intergenerational transfer of oyster microbes is possible, they do not necessarily fit the current definition of the ‘consistent microbiome transmission’ assumption of the hologenome theory. Further testing is required to determine whether the patterns reported here persist across other marine broadcast spawning marine organisms.

## Supplementary Information

**Additional file 1**. Supplementary information for Microbiomes of the Sydney Rock Oyster are acquired through both vertical and horizontal transmission.

## Data Availability

The datasets generated and analyzed during the current study are available in the NCBI SRA database under BioProject ID no. PRJNA 761,332. https://www.ncbi.nlm.nih.gov/bioproject/761332.

## Abbreviations

USD: United States Dollars
NSW: New South Wales
DPI: Department of Primary Industries
PSFI: Port Stephens Fisheries Institute
PSU: Practical salinity unit
PCR: Polymerase chain reaction
ASV: Amplicon sequence variant
NMDS: Non-metric multidimensional scaling
PERMANOVA: Permutational multivariate analysis of variance
