# Supplementary material for: Microbiomes of the Sydney Rock Oyster are acquired through both vertical and horizontal transmission
Source: Anim Microbiome. 2022 May 19;4:32. doi: 10.1186/s42523-022-00186-9 (PMC9118846; doi:10.1186/s42523-022-00186-9)
Supplement: Supplementary file 1 — Additional file 1. Supplementary information for Microbiomes of the Sydney Rock Oyster are acquired through both vertical and horizontal transmission. [file 42523_2022_186_MOESM1_ESM.pdf]

Supplemental information for:

Microbiomes of the Sydney Rock Oyster are acquired through both vertical and horizontal transmission

Author names: Andrea Unzueta-Martínez[1,6], Elliot Scanes[2,3], Laura M. Parker[4], Pauline M. Ross[2], Wayne O'Connor[5], Jennifer L. Bowen[1]

Table S1. Statistical analysis of the effects of adult tissues, gamete type, and larval stage on Sorensen-Dice dissimilarities of oyster-associated microbial communities. (A) Results of a permutational test for homogeneity of group dispersions (betadisper). (B) Results of PERMANOVA (Adonis), using 999 permutations. (C) Results of pairwise comparisons (pairwiseAdonis).

A. Homogeneity of group dispersions test:

Factor	DF	Sum of Squares	Mean Squares	F	Pr(>F)	significance
Adult tissues, laval stage, and gametes	10	0.063081	0.0063081	2.7811	0.00511	**
Residuals	84	0.19053	0.0022682			

C. PEMANOVA:

Factor	Df	Sum of Squares	Mean Squares	F Model	R2	Pr(>F)	significance
Adult tissues, laval stage, and gametes	10	11.035	1.1035	2.9159	0.25768	0.001	***
Residuals	84	31.787	0.378416667		0.74232		
Total	94	42.822			1		

D. Pairwise comparisons of PERMANOVA:

Pairs			Df	SumsOfSqs	F.Model	R2	p.value	p.adjusted	significance
Ds_day1	vs	Ds_day3	1	0.7505393	2.16809	0.08614441	0.001	0.021	.
Ds_day1	vs	eyedpedi_day29	1	1.1148419	3.316702	0.15559171	0.001	0.021	.
Ds_day1	vs	spat_day34	1	1.061429	2.989227	0.14241722	0.001	0.021	.
Ds_day1	vs	umbo_day15	1	1.166573	3.420755	0.14007572	0.001	0.021	.
Ds_day1	vs	eggs	1	0.9104951	2.566942	0.1311877	0.001	0.021	.
Ds_day1	vs	gill	1	2.2569566	6.96285	0.24040626	0.001	0.021	.
Ds_day1	vs	gonad	1	1.6398473	4.490522	0.16951429	0.001	0.021	.
Ds_day1	vs	mantle	1	1.7275726	4.816204	0.1796005	0.001	0.021	.
Ds_day1	vs	stomach	1	1.7001327	4.818139	0.18661837	0.001	0.021	.
Ds_day1	vs	sperm	1	1.1501343	3.259619	0.16089241	0.001	0.021	.
Ds_day3	vs	eyedpedi_day29	1	0.9494599	2.586022	0.14704984	0.001	0.021	.
Ds_day3	vs	spat_day34	1	0.8761088	2.247027	0.1302849	0.001	0.021	.
Ds_day3	vs	umbo_day15	1	0.9163768	2.492218	0.12161779	0.001	0.021	.
Ds_day3	vs	eggs	1	0.8509958	2.17137	0.13427247	0.001	0.021	.
Ds_day3	vs	gill	1	1.9049167	5.493786	0.22429304	0.001	0.021	.
Ds_day3	vs	gonad	1	1.3962465	3.541464	0.15710888	0.001	0.021	.
Ds_day3	vs	mantle	1	1.4712401	3.80407	0.16681541	0.001	0.021	.
Ds_day3	vs	stomach	1	1.4568705	3.818792	0.17502309	0.001	0.021	.
Ds_day3	vs	sperm	1	0.9972382	2.559242	0.15455067	0.001	0.021	.
eyedpedi_day29	vs	spat_day34	1	0.5425834	1.37825	0.12113024	0.024	1	
eyedpedi_day29	vs	umbo_day15	1	0.6611302	1.826009	0.12316257	0.001	0.021	.
eyedpedi_day29	vs	eggs	1	0.7600548	1.913352	0.17532215	0.002	0.11	
eyedpedi_day29	vs	gill	1	1.4921975	4.467306	0.24190348	0.001	0.021	.
eyedpedi_day29	vs	gonad	1	1.0927931	2.742176	0.16378851	0.001	0.021	.
eyedpedi_day29	vs	mantle	1	1.171469	3.016677	0.1772777	0.001	0.021	.
eyedpedi_day29	vs	stomach	1	1.1452102	3.004401	0.18772341	0.001	0.021	.
eyedpedi_day29	vs	sperm	1	0.8992472	2.283922	0.20240496	0.001	0.021	.
spat_day34	vs	umbo_day15	1	0.7447548	1.917947	0.12856641	0.001	0.021	.
spat_day34	vs	eggs	1	0.6598677	1.516424	0.14419575	0.003	0.165	
spat_day34	vs	gill	1	1.2779792	3.565819	0.20299761	0.001	0.021	.
spat_day34	vs	gonad	1	0.900407	2.129206	0.13200937	0.001	0.021	.
spat_day34	vs	mantle	1	0.9678319	2.345113	0.14347485	0.001	0.021	.
spat_day34	vs	stomach	1	0.9836047	2.41421	0.15662234	0.001	0.021	.
spat_day34	vs	sperm	1	0.7510169	1.739917	0.16200468	0.004	0.22	
umbo_day15	vs	eggs	1	0.8016822	2.052793	0.14607725	0.002	0.11	
umbo_day15	vs	gill	1	1.7410025	5.113853	0.23125111	0.001	0.021	.
umbo_day15	vs	gonad	1	1.2077541	3.068831	0.1529153	0.001	0.021	.
umbo_day15	vs	mantle	1	1.3109579	3.403585	0.16681309	0.001	0.021	.
umbo_day15	vs	stomach	1	1.2845592	3.387907	0.17474331	0.001	0.021	.
umbo_day15	vs	sperm	1	0.955566	2.463424	0.17032097	0.002	0.11	
eggs	vs	gill	1	1.0282107	2.870901	0.18089086	0.001	0.021	.
eggs	vs	gonad	1	0.6233858	1.457884	0.10083658	0.001	0.021	.
eggs	vs	mantle	1	0.704139	1.690078	0.11504897	0.003	0.165	
eggs	vs	stomach	1	0.7660537	1.86279	0.13437339	0.002	0.11	
eggs	vs	sperm	1	0.5577293	1.266438	0.13666939	0.026	1	
gill	vs	gonad	1	1.163073	3.143447	0.14867239	0.001	0.021	.
gill	vs	mantle	1	0.891905	2.463283	0.12037575	0.001	0.021	.
gill	vs	stomach	1	1.2713987	3.580745	0.17398518	0.001	0.021	.
gill	vs	sperm	1	1.0234818	2.877207	0.18121617	0.001	0.021	.
gonad	vs	mantle	1	0.5506568	1.33578	0.06908332	0.023	1	
gonad	vs	stomach	1	0.6428328	1.574906	0.08478675	0.002	0.11	
gonad	vs	sperm	1	0.7363256	1.731846	0.11755799	0.001	0.021	.
mantle	vs	stomach	1	0.8247523	2.06298	0.10821918	0.001	0.021	.
mantle	vs	sperm	1	0.74155	1.790308	0.12104602	0.001	0.021	.
stomach	vs	sperm	1	0.8506972	2.081934	0.14784432	0.001	0.021	.

Table S2. Statistical analysis of the effects of the environment (feed and tank water) and larval stage on Sorensen-Dice dissimilarities of oyster-associated microbial communities. (A) Results of a permutational test for homogeneity of group dispersions (betadisper). (B) Results of PERMANOVA (Adonis), using 999 permutations. (C) Results of pairwise comparisons (PairwiseAdonis).

A. Homogeneity of group dispersions test:

Factor	DF	Sums of Squares	Mean Square	F	Pr(>F)	significance
Larval stage and environmnet	6	0.019089	0.0047722	1.5505	0.2057	NS
Residuals	41	0.12619	0.0030778			

B. PERMANOVA:

Factor	DF	Sum of Squares	Mean Squares	F Model	R2	Pr(>F)	significance
Larval stage and environmnet	6	6.476	1.07933	2.9731	0.23522	0.001	***
Residuals	58	21.056	0.36303	0.76478			
Total	64	27.532					

C. Pairwise comparisons:

Pairs			Df	SumsOfSqs	F.Model	R2	p.value	p.adjusted	significance
Ds_day1	vs	Ds_day3	1	0.9288594	6.030348	0.2151364	0.001	0.021	.
Ds_day1	vs	eyedpedi_day29	1	1.9201005	11.546303	0.4044763	0.001	0.021	.
Ds_day1	vs	spat_day34	1	2.2119505	13.669189	0.4456978	0.001	0.021	.
Ds_day1	vs	umbo_day15	1	2.5292943	15.953654	0.4437283	0.001	0.021	.
Ds_day1	vs	algae	1	3.6493241	24.462861	0.5501864	0.001	0.021	.
Ds_day1	vs	water	1	2.3723006	12.466037	0.3616905	0.001	0.021	.
Ds_day3	vs	eyedpedi_day29	1	1.4023679	4.953297	0.2482445	0.002	0.042	.
Ds_day3	vs	spat_day34	1	1.3571353	4.880977	0.2455099	0.001	0.021	.
Ds_day3	vs	umbo_day15	1	1.4747988	5.782836	0.2431517	0.001	0.021	.
Ds_day3	vs	algae	1	2.0595343	8.41904	0.3186732	0.001	0.021	.
Ds_day3	vs	water	1	1.3130333	4.684078	0.1897611	0.001	0.021	.
eyedpedi_day29	vs	spat_day34	1	0.7289794	2.019873	0.1680445	0.003	0.063	
eyedpedi_day29	vs	umbo_day15	1	0.7511543	2.423743	0.1571436	0.001	0.021	.
eyedpedi_day29	vs	algae	1	1.4982533	5.070026	0.2805766	0.002	0.042	.
eyedpedi_day29	vs	water	1	1.2023961	3.575214	0.1924723	0.001	0.021	.
spat_day34	vs	umbo_day15	1	0.9916723	3.26141	0.2005613	0.001	0.021	.
spat_day34	vs	algae	1	1.0908377	3.765933	0.2246182	0.001	0.021	.
spat_day34	vs	water	1	1.2949102	3.909255	0.2067376	0.001	0.021	.
umbo_day15	vs	algae	1	1.4933415	5.708722	0.2629691	0.001	0.021	.
umbo_day15	vs	water	1	1.6610045	5.548505	0.2356203	0.001	0.021	.
algae	vs	water	1	2.2620089	7.828144	0.3030858	0.001	0.021	.

Table S3. Statistical analysis of the effects of family on Sorensen-Dice dissimilarities of oyster-associated microbial communities. (A) Results of a permutational test for homogeneity of group dispersions (betadisper). (B) Results of PERMANOVA (Adonis), using 999 permutations.

A. Homogeneity of group dispersions test:

Factor	DF	Sums of Squares	Mean Square	F	Pr(>F)	significance
Family	4	0.019089	0.0047722	1.5505	0.204	NS
Residuals	41	0.12619	0.0030778			

B. PERMANOVA:

Factor	DF	Sum of Squares	Mean Squares	F Model	R2	Pr(>F)	significance
Family	4	1.7	0.425	1.0601	0.09373	0.204	NS
Residuals	41	16.437	0.400902439		0.90627		
Total	45	18.137			1		

Table S4. Taxonomic information, identified by comparison with the Silva database, of 30 amplicon sequence variants (ASVs) with most important contributions to a Random Forest regression model trained to predict larval stage from microbial community composition.

Kingdom	Phylum	Class	Order	Family	Genus	ASV	Sequence Md5 hash ID
Bacteria	Bacteroidetes	Bacteroidia	Flavobacteriales	Flavobacteriaceae	Kordia	ASV_2	59ccf8d304653395c95d4537eca76a6e
Bacteria	Proteobacteria	Gammaproteobacteria	Alteromonadales	Alteromonadaceae	Aestuariibacter	ASV_4	bbe8d993a60d3448b591fd119c28452780599458
Bacteria	Proteobacteria	Alphaproteobacteria	Sphingomonadales	Sphingomonadaceae	Sphingorhabdus	ASV_12	04cff81844348932067bc099bbaeeba0476b3c3a
Bacteria	Proteobacteria	Gammaproteobacteria	Cellvibrionales	Cellvibrionaceae	Aestuariicella	ASV_15	b7f23f4bf88fb0b2c6b82824d33e8782a893083c
Bacteria	Proteobacteria	Gammaproteobacteria	Alteromonadales	Marinobacteraceae	Marinobacter	ASV_19	de577eac38335e589c5c9ed66d23aaf7b38bcaf1
Bacteria	Proteobacteria	Alphaproteobacteria	Rhizobiales	Stappiaceae	Labrenzia	ASV_25	87ebfbdb7a84e4677d7b60df7edfa45e62d4def3;size=74339
Bacteria	Proteobacteria	Alphaproteobacteria	Rhodobacterales	Rhodobacteraceae	Antarctobacter	ASV_26	b6e45e9e6bffa1f2ffb7d6375dfde7da0e4b83af;size=69986
Bacteria	Proteobacteria	Gammaproteobacteria	Alteromonadales	Alteromonadaceae	Aestuariibacter	ASV_31	119464842cb5358793b492a5d082121831b5b1e0
Bacteria	Proteobacteria	Alphaproteobacteria	Rhodobacterales	Rhodobacteraceae	Nautella	ASV_33	637e056b57a8f2cd33312ba5ef812776730645e5
Bacteria	Proteobacteria	Gammaproteobacteria	Cellvibrionales	Spongiibacteraceae	Spongiibacter	ASV_43	cdea786612b5415a97ad2e49997575e7ee7b3d9a
Bacteria	Proteobacteria	Gammaproteobacteria	Oceanospirillales	Oleiphilaceae	Oleiphilus	ASV_59	073b0359e8534fe71292b4db9a95727fba1a1c48
Bacteria	Proteobacteria	Gammaproteobacteria	Alteromonadales	Marinobacteraceae	Marinobacter	ASV_63	f126fbf6e0a0c3e321944a34844d40ef72377d1b
Bacteria	Proteobacteria	Gammaproteobacteria	Oceanospirillales	Oleiphilaceae	Oleiphilus	ASV_68	692ff2875fb15e9780efd4d80383dc4ef150fa5c
Bacteria	Proteobacteria	Alphaproteobacteria	Sphingomonadales	Sphingomonadaceae	NA	ASV_78	f9bb73d48255f7bfa615ebea722862e7b6cc80ac
Bacteria	Proteobacteria	Gammaproteobacteria	Alteromonadales	Marinobacteraceae	Marinobacter	ASV_89	b380ab07413b5452cfcbbb245ec422a8313a44f5
Bacteria	Proteobacteria	Gammaproteobacteria	Alteromonadales	Marinobacteraceae	Marinobacter	ASV_94	4e43625e983c08d5543a79836f5a322acc57a0c9
Bacteria	Proteobacteria	Gammaproteobacteria	Oceanospirillales	Saccharospirillaceae	Bermanella	ASV_121	ea3e7cd24ffc41e546033dce18ce1a32ad70063a
Bacteria	Proteobacteria	Gammaproteobacteria	Salinisphaerales	Salinisphaeraceae	Salinisphaera	ASV_123	8aa0f891e91dcafdbb9142b8406571d18f03b9ad
Bacteria	Proteobacteria	Alphaproteobacteria	Rhodobacterales	Rhodobacteraceae	Ruegeria	ASV_125	97fbcf509eef3b8d527956b26382dcbb969d4891
Bacteria	Proteobacteria	Gammaproteobacteria	Alteromonadales	Alteromonadaceae	Aestuariibacter	ASV_130	9f79a9ab9f3191bb6ff66eb16a5d6eea8a658bf3
Bacteria	Proteobacteria	Alphaproteobacteria	Rhodobacterales	Rhodobacteraceae	Thalassobius	ASV_159	5d5ce1621ae721b880a6fa4e9c1c4d40f034a8be
Bacteria	Proteobacteria	Gammaproteobacteria	Cellvibrionales	Halieaceae	Pseudohaliea	ASV_174	25dd1bf775f3aece7c10551f3b130720c571223f
Bacteria	Proteobacteria	Gammaproteobacteria	Alteromonadales	Alteromonadaceae	XY-R5	ASV_198	6eed584613ef3a2e903c89d61c680029951a4d23
Bacteria	Proteobacteria	Gammaproteobacteria	Alteromonadales	Alteromonadaceae	NA	ASV_249	ecdfbcb6619a79417e1de4ef483bc15bb263d5c7
Bacteria	Proteobacteria	Gammaproteobacteria	Alteromonadales	Marinobacteraceae	Marinobacter	ASV_272	b94019d443210537ad520a471e96c4d64ab3b49a
Bacteria	Proteobacteria	Alphaproteobacteria	Caulobacterales	Hyphomonadaceae	Maricaulis	ASV_281	11dc05b978ff8b8a15e3aa9231765edbee20afc8
Bacteria	Proteobacteria	Alphaproteobacteria	Caulobacterales	Hyphomonadaceae	Hyphomonas	ASV_336	513a0b5fae74775db0bf5e8f115d3104bdd4695
Bacteria	Proteobacteria	Deltaproteobacteria	Myxococcales	Haliangiaceae	Haliangium	ASV_379	a595b48198e077d6ff54f1bfe5c17efa5766277b
Bacteria	Proteobacteria	Alphaproteobacteria	Caulobacterales	Hyphomonadaceae	Hyphomonas	ASV_527	b3bd97486e864e99eef2b8f461e49ec4b4414c5d
Bacteria	Proteobacteria	Gammaproteobacteria	Cellvibrionales	Spongiibacteraceae	BD1-7_clade	ASV_555	8c8a73b022f33c1360db536f38e6055a5f1a938a

Table S5. Taxonomic information, identified by comparison with the Silva database, of 14 ASVs identified as core members (present at greater than 1% abundance in more than 50% of samples) in the eggs, sperm, and all larval stages. This analysis excluded all ASVs detected in tank water and algae samples with a relative abundance greater than 1%.

Phylum	Class	Order	Family	Genus	ASV	Sequence Md5 hash ID
Proteobacteria	Gammaproteobacteria	Vibrionales	Vibrionaceae	<NA>	ASV_11	ae0a06a8a1f235c0059aa19ef8ab090f37abc25e
Proteobacteria	Alphaproteobacteria	Rhodobacterales	Rhodobacteraceae	Nautella	ASV_33	637e056b57a8f2cd33312ba5ef812776730645e5
Proteobacteria	Gammaproteobacteria	Cellvibrionales	Spongiibacteraceae	Spongiibacter	ASV_43	cdea786612b5415a97ad2e49997575e7ee7b3d9a
Proteobacteria	Gammaproteobacteria	Alteromonadales	Pseudoalteromonadaceae	Pseudoalteromonas	ASV_44	baa02e16825b5f16263863ccfc9388065defc9bb
Proteobacteria	Gammaproteobacteria	Oceanospirillales	Oleiphilaceae	Oleiphilus	ASV_68	692ff2875fb15e9780efd4d80383dc4ef150fa5c
Bacteroidetes	Bacteroidia	Flavobacteriales	Flavobacteriaceae	Lutaonella	ASV_83	ca080168f52fba62bc6caf5870aafb4375bfdd36
Proteobacteria	Gammaproteobacteria	Oceanospirillales	Saccharospirillaceae	Bermanella	ASV_121	ea3e7cd24ffc41e546033dce18ce1a32ad70063a
Proteobacteria	Gammaproteobacteria	Oceanospirillales	Oleiphilaceae	Oleiphilus	ASV_141	4064c3a4a190731797c92800e5ea21a66b0794ab
Proteobacteria	Alphaproteobacteria	Rhodobacterales	Rhodobacteraceae	<NA>	ASV_150	ee41c2d9cf1bfddedb846f847fe56d21b44a7cc8
Proteobacteria	Alphaproteobacteria	Rhodobacterales	Rhodobacteraceae	Thalassobius	ASV_159	5d5ce1621ae721b880a6fa4e9c1c4d40f034a8be
Proteobacteria	Gammaproteobacteria	Alteromonadales	Alteromonadaceae	XY-R5	ASV_179	087a2484e3677d0922a89c3d7b7dfbde2734e9d7
Proteobacteria	Alphaproteobacteria	Rhodobacterales	Rhodobacteraceae	Tropicimonas	ASV_354	c6924e36d9992d8a541978ff901b0237a5b111cb
Proteobacteria	Gammaproteobacteria	Alteromonadales	Marinobacteraceae	Marinobacter	ASV_263	fbfbe83b4dd1fbe7b8edf71a6845a39e5ca43328
Proteobacteria	Gammaproteobacteria	Alteromonadales	Marinobacteraceae	Marinobacter	ASV_272	b94019d443210537ad520a471e96c4d64ab3b49a

Table S6. Mann-Whitney U test results of comparing the percentage of shared ASVs between larvae and their parent eggs and larvae and their parent sperm for each larval stage. This analysis excluded all ASVs detected in tank water and algae samples with a relative abundance greater than 1%.

Comparison day	Kruskal-Wallis chi-squared	df	p-value	significance
**DAY 1**				
eggs vs. sperm	3.6573	1	0.0548	.
**DAY 3**				
eggs vs. sperm	4.2014	1	0.0404	.
**DAY 15**				
eggs vs. sperm	5.4087	1	0.0200	.
**Day 29**				
eggs vs. sperm	9.504	1	0.0021	***
**Day 34**				
eggs vs. sperm	2.2331	1	0.1251	NS

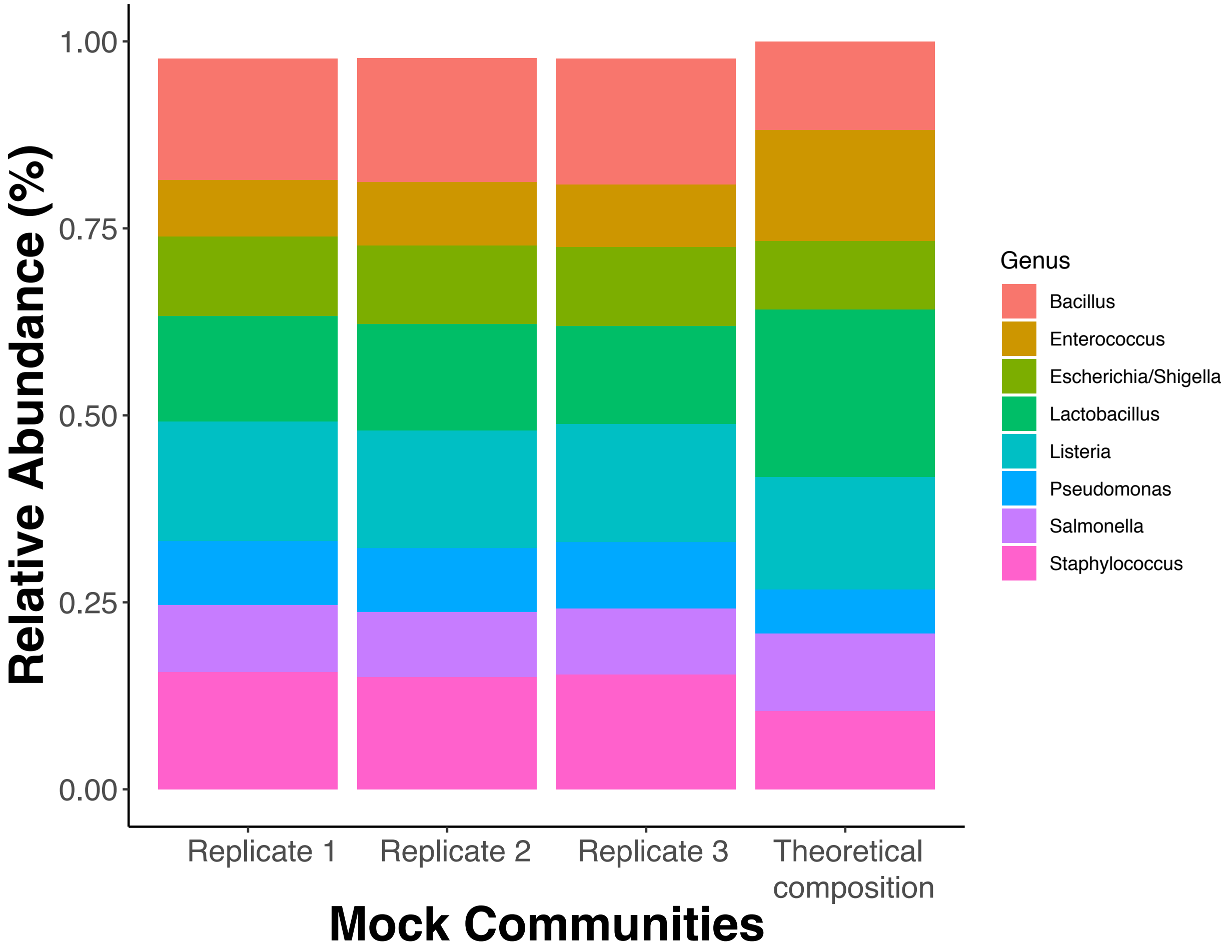

Figure S1. Stacked bat plot of the relative abundance of bacterial genera comprising mock community replicates and the theoretical composition. Relative abundance was calculated within each sample and ASVs that made up less that 1% of the sample were excluded.

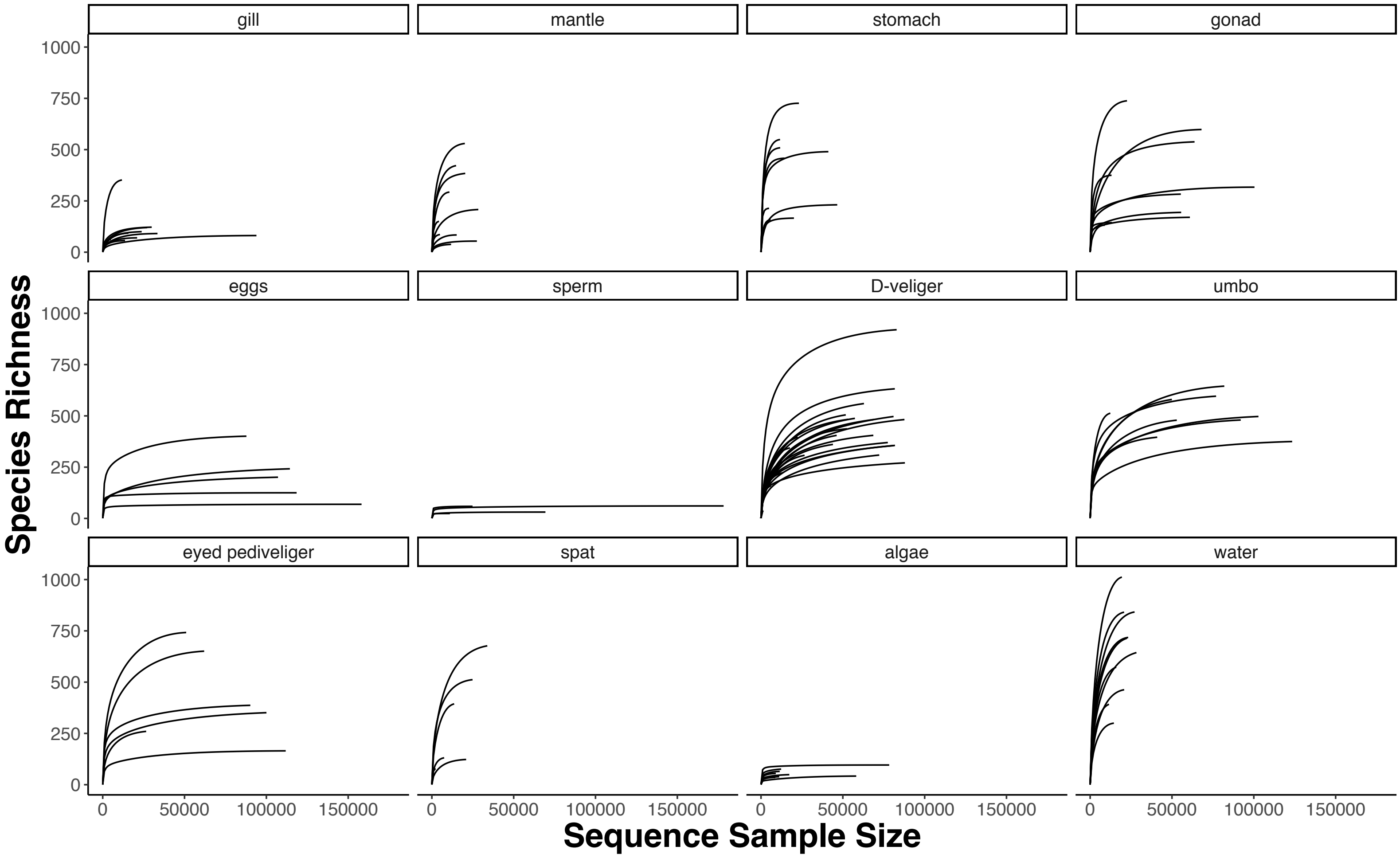

Figure S2. Rarefaction curves of the number of ASVs versus the number of sequences in each sample grouped by sample type.

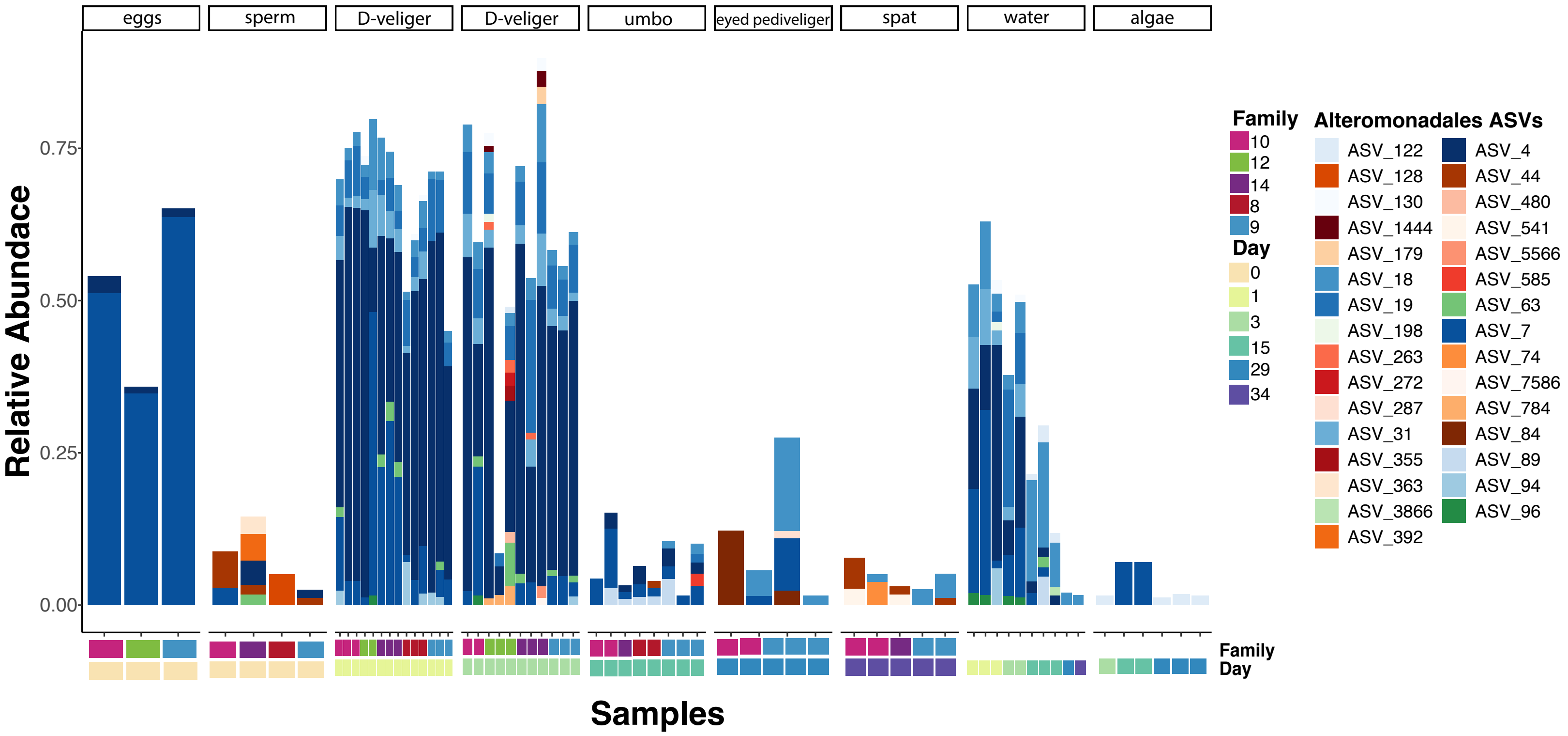

Figure S3. Stacked bar plot of the relative abundance of Alteromonadales ASVs associated with oyster tissues (gametes and larvae) and their environment (water and algae). Relative abundance was calculated within each sample. Warm ASV colors (reds and oranges), represent Alteromonadales ASVs that were detected on oyster tissues but not their environment, while cool ASV colors (blues and greens) represent Alteromonadales ASVs that were detected on oyster tissues and their environment. Samples that contained no Alteromonadales ASVs are not depicted in the figure.
